# Nonverbal expressions of shame predict suicidal ideation among rurally-situated, but not urban situated, lesbian, gay, bisexual, transgender, and queer (LGBTQ) adults

**DOI:** 10.1371/journal.pmen.0000129

**Published:** 2025-01-30

**Authors:** Mollie A. Ruben, Michelle A. Stage, Abigail W. Batchelder, Craig Gilbert, Jillian C. Shipherd, Nicholas A. Livingston, Adele E. Weaver, Danielle S. Berke

**Affiliations:** 1 Department of Psychology, University of Rhode Island, Kingston, Rhode Island, United States of America; 2 Department of Psychiatry, Boston University Chobanian & Avedisian School of Medicine, Boston, Massachusetts, United States of America; 3 The Fenway Institute, Fenway Health, Boston, Massachusetts, United States of America; 4 The Graduate Center, City University of New York, New York, New York, United States of America; 5 Department of Veterans Affairs, LGBTQ+ Health Program, Washington, District of Columbia, United States of America; 6 Behavioral Science Division, National Center for PTSD, VA Boston Healthcare System, Boston, Massachusetts, United States of America; 7 Hunter College of the City University of New York, New York, New York, United States of America; The University of Waikato, NEW ZEALAND

## Abstract

In the United States (US), lesbian, gay, bisexual, transgender, and queer (LGBTQ) people experience disproportionate rates of suicidality associated with minority stress. This study aimed to investigate whether nonverbal expressions of experienced stigma (i.e., shame) predicted suicidal ideation among LGBTQ individuals with a focus on location-based disparities (comparing those living in a more rural setting to those living in a more urban setting). More specifically, we examined whether nonverbal expressions of shame predicted suicidal ideation three months later and whether this relationship was moderated by region. LGBTQ individuals (*N* = 133) from one rurally-situated and one urban location were videorecorded while talking about a time they felt bad about their LGBTQ identity in an observational, prospective (two-time point) design. Recordings were coded for the intensity of nonverbal expressions of shame (shoulders slumped, chest narrowed). Participants also completed several self-report measures including state shame and suicidal ideation at both the time of the recording and three months later. Moderation analyses revealed that for LGBTQ adults living in more rural settings, nonverbal shame, predicted increased suicidal ideation three months later (*B*_*std*_ = 0.64, *p* = .005), and this was not the case for those living in more urban settings (*B*_*std*_ = -0.08, *p* = 0.70). Self-reported shame did not predict suicidal ideation for LGBTQ adults from more rural or urban locations. These findings highlight the importance of recognizing nonverbal cues in context (i.e., in locations with more structural stigma) when assessing mental health risks and when shaping interventions for LGBTQ populations.

## Introduction

Lesbian, gay, bisexual, transgender, and queer (LGBTQ) individuals living in more rural settings not only experience more societal and interpersonal stigma [1], but they also experience poorer outcomes related to stigma compared to their urban counterparts [2–4]. Cultural dynamics within rural communities often reinforce cisnormative and heteronormative hierarchies and policies, increasing anti-LGBTQ attitudes and further marginalizing LGBTQ individuals [5]. According to Meyer’s minority stress model [6, 7], the mental and physical health disparities LGBTQ populations experience can be explained by the stigma that LGBTQ people face daily [8]. Given that stigma can be embodied and directly impact health, it is plausible that there may be nonverbal signs of experienced stigma that predict health outcomes for LGBTQ individuals. This may be especially true among those living in locations where stigma is more prevalent (e.g., more rurally-situated locations). Therefore, the purpose of the current research was to test whether nonverbal expressions of stigma, namely shame, predicted suicidal ideation among LGBTQ individuals and whether location moderated this relationship.

### Stigma and health

People who are stigmatized have (or are believed to have) an attribute that marks them as different and leads them to be devalued in the eyes of others [9, 10]. Importantly, stigma is relationship- and context-specific; it does not reside within the person but in a social context. In the current work, we focus on structural and individual stigma [11].

Structural stigma includes societal-level conditions, cultural norms, and institutional policies that constrain the opportunities, resources, and wellbeing of the stigmatized. Examples of structural stigma include state laws that ban gender affirming care or employers denying health insurance benefits to same-sex couples. Sexual minorities living in states *without* policies that protected them against hate crimes, housing, or employment discrimination report higher levels of community stigma and experience significantly higher rates of anxiety disorders, depression, and post-traumatic stress disorder (PTSD) than those who lived in states with protections [12–14]. Like this past work, we hypothesized that where people live would play a role in the relationship between nonverbal expressions of shame and suicidal ideation.

Though discrimination against LGBTQ individuals is not unique to rural areas, the impact may be different. Factors such as pressure to conform to rural cultural norms and fear of rejection from family and peers may intensify feelings of stigma [15], especially when such communities are tightly interwoven and rejection in one area of life (e.g., faith) often ripples into others (e.g., work or school) [16]. Additionally, rural LGBTQ individuals report more loneliness and less community connectedness than urban LGBTQ individuals [3]. Finally, rural settings have an established lack of access and funding related to health services combined with mental health stigma [17, 18]. These factors may compound the experiences of stigma experienced by LGBTQ people living in more rural settings compared to those living in more urban settings [19, 20].

Individual stigma refers to the psychological processes in which individuals engage in and respond to stigma, such as the internalization of negative societal views about one’s group. For example, LGBTQ individuals may internalize societal messages that their sexual orientation or gender is wrong. For example, a gay man might feel shame about his sexuality due to repeated exposure to anti-LGBTQ rhetoric from family, religious institutions, or the media. One of these individual level stigma processes that is less well understood among LGBTQ populations is shame, yet it is well-documented that LGBTQ individuals experience shame [21]. Shame is defined as a painful affective response to the experience of believing one is flawed and unworthy of belonging [22]. Shame can occur when individuals blame themselves for aspects of the self that are stable, uncontrollable, and immutable [23, 24]. It is argued that because shame is linked to one’s core identity, it is one of the most powerful and significant affective experiences [6, 7]. Shame threatens social bonds and leads to withdrawal, isolation, and concealment [6, 7]. Self-reported shame is linked to suicidal ideation, depression, anxiety, eating disorders, chronic anger, heightened cortisol reactivity, and poor immune system functioning [25–30]. Several distinct theories suggest that feelings of shame contribute to suicidality [31–35]. For example, in the Interpersonal Theory of Suicide, perceived burdensome and thwarted belongingness are critical factors contributing to suicidal ideation [34]. Shame directly contributes to both factors by leading people to believe they are a burden to others and do not belong. Additionally, because shame typically involves viewing the self as flawed and provokes a desire to escape or hide, suicide may represent “the ultimate escape” [29].

Sexual minorities are six times more likely than their heterosexual counterparts to attempt suicide in their lifetime [36, 37]. Among transgender adults in the US, 81% have thought about suicide and 42% have attempted suicide [38]. These disparities in suicide rates also vary based on region such that those in more rural settings report higher suicidal ideation and attempts [39]. While the minority stress model and a growing body of research has identified stigma-related processes as important explanations of LGBTQ health disparities [7, 13], advancements in this area are hampered by an overreliance on self-report measures. Shame is thought to be experienced implicitly, making it challenging for individuals to accurately self-report [40]. Nonverbal behaviors of state shame may reflect more accurate momentary feelings that may better predict future health and wellbeing. This may be especially true for nonverbal indicators of suicidal ideation, as research suggests that fewer than half of people with suicidal ideation verbally disclose these thoughts [41]. Given that nonverbal behaviors are harder to control than verbal behaviors, they may be a better indicator of suicidal ideation, especially among populations from rural settings where LGBTQ stigma is more prevalent [42].

Expressions associated with shame can be assessed from spontaneous nonverbal behaviors. For example, among Olympic athletes who lost a medal match, they displayed more shoulders slumped and chest narrowed than those who won [43]. In addition, past work has shown that children show these same collapsed postures in response to experimentally manipulated failures [44, 45]. Researchers have suggested an evolutionary origin to these behaviors as they require individuals to place themselves physically beneath others which may signal appeasing onlookers or awareness of one’s “transgression” to in order to reduce conflict, maintain one’s reputation, and communicate an acceptance of social norms [46–48].

In several other studies, brief behavioral manifestations of nonverbal shame (i.e., more shoulders slumped and chest narrowed) were linked to important identity-related health outcomes. For example, in one sample, 10-second coded nonverbal expressions of shame talking about one’s last drink predicted relapse and severity of relapse 6 months later among participants with alcohol use disorder [49]. In another study among people living with HIV, coded nonverbal expressions of shame while talking about one’s HIV diagnosis were related to more distancing as a coping method [50]. Importantly, in both studies, nonverbal shame was consistently *unrelated* to self-reported measures of shame, suggesting a unique internalized construct that self-reported shame does not tap into.

### Current study

Thus, we tested for the first time the relationship between brief, nonverbal expressions of shame and suicidal ideation among an LGBTQ sample that varied in location-based stigma. We hypothesized that higher levels of expressed nonverbal shame while talking about one’s LGBTQ identity at Time 1 (T1) would predict increased suicidal ideation at 3-month follow-up (T2), and that this relationship would be moderated by or stronger for those living in more rural locations compared to more urban locations given evidence that structural stigma impacts rural LGBTQ adults differently than urban LGBTQ adults [2–4]. We aimed to examine change in suicidal ideation (and predict suicidal ideation 3 months later) because we conceptualize nonverbal expressions of shame as an observable warning sign or risk factor predictive of future behavior as past work suggests [49].

## Methodology

### Participants and procedure

The study protocol was approved by the Institutional Review Boards at the University of Maine and Hunter College and all participants provided written informed consent. Individuals who self-identified as LGBTQ were recruited from the University of Maine and Hunter College participant pool, LGBTQ-friendly social media websites, listservs, and flyers to participate in a two time-point study (N = 133). Approximately half (n = 68) were recruited from a more rural location (central Maine) and n = 65 were recruited from a more urban location (New York City). Participants predominantly identified as women, lesbian, gay, or bisexual, non-Hispanic or non-Latine White. Participants ranged in age from 18–53 though tended to be young adults (*M*_age_ = 22.63, *SD*_age_ = 6.66).

At both sites, participants were either granted course credit or paid $15 for completing T1, during which they completed a series of questionnaires and had a videotaped interaction either in person or on Zoom. Data collection for this study started in October 2019 and was interrupted by the 2020 COVID-19 pandemic. When the pandemic started, we transitioned from in person laboratory sessions to Zoom sessions with self-view off. We tested whether there were any differences in self-reports of shame or nonverbal expressions of shame for those who conducted the study in person vs. online and found no significant or meaningful differences. Therefore, we collapsed channel by which participants completed the study. Data collection ended in January 2021.

All participants were paid with a $10 Amazon.com gift card for completing a 3-month follow-up assessment (T2). This time frame was chosen based on prior research suggesting that the first 10-seconds of discussing feeling badly about one’s identity represents an important and meaningfully predictive time period for health outcomes [49]. In our rural sample, n = 48 (74%) returned for T2; in our urban sample, n = 51 (78%) returned for T2.

A power analysis conducted using G*Power including predictors nonverbal shame, rurality of residence, and the interaction between nonverbal shame and rurality indicated that 89 participants would be required to achieve 95% power to detect small associations (*f*^*2*^ = 0.15) between the predictors and outcomes using an alpha level of 0.05 (two-tailed). The final sample of N_T1_ = 133 exceeded this target even after attrition from T1 to T2 (N_T2_ = 99) [51].

### Measures

#### Nonverbal expressions of shame

At T1, participants were videorecorded while they responded to a prompt asking them to talk for a few minutes about a time they felt bad because of their LGBTQ identity. If participating on Zoom, participant’s self-view was turned off prior to the recording and they were given instructions to make sure their body torso and face were in frame. All participants completed the interview without anyone else present other than the trained experimenter.

Four reliable research assistants (blind to hypotheses) were trained to watch all videos (without audio) and code the first 10 seconds of nonverbal behavior. The first 10 seconds were chosen because this is when we expected participants would express the strongest and most spontaneous expressions, as they did not know the questions ahead of time, consistent with previous nonverbal shame work [49]. Behavioral coding with numerous coders is labor intensive, and participants spoke at varied lengths, therefore we also chose this timeframe to standardize coding and reduce coder burden.

Shame displays were coded using Tracy and Matsumoto’s previously validated shame behavioral coding scheme [43]. This scheme involves coding the degree to which participants displayed chest narrowed (ICC = 0.80) and shoulders slumped (ICC = 0.83) from 0 (“not at all present”) to 1 (“visible but very mild intensity”) to 5 (“extreme intensity”). These behaviors were combined into a mean shame display scale (α = 0.77). Head-tilt down, often treated as part of the nonverbal shame display, could not be reliably assessed, because a participant’s height, chair height, and computer orientation impacted the orientation of their head to the camera. However, head-tilt down is not essential to the display, whereas the broader body movements examined in the current work tend to be more reliably related to shame [43]. Higher codes reflected more nonverbal expressions of shame.

#### Self-reported shame

After completing the videorecorded narrative, participants completed the State Shame and Guilt Scale [52], a validated self-report measure of momentary shame and guilt experiences (α = 0.75) [28]. Participants were asked to answer items based on *how they are feeling right now*. An example item includes, “I want to sink into the floor and disappear”. Participants responded on a scale from *not feeling this way at all* (1) to *feeling this way very strongly* (5). Higher scores reflected more self-reported state shame.

Suicidal ideation

At both timepoints, participants completed the Depressive Symptom Index Suicidality Subscale (DSI-SS) which measures the frequency and intensity of suicidal ideas and impulses in the past two weeks (α = 0.94 for T1 and α = 0.89 for T2) [53]. Higher scores reflected more suicidal ideation.

#### Rurality

Current residence was used to stratify our sample into more rural (those currently living in central Maine) vs. more urban residence (those currently living in New York City).

### Statistical analyses

First, descriptive analyses were conducted to describe the demographic characteristics of the sample. We compared rural vs. urban participants on demographic characteristics, nonverbal expressions of shame, and self-reported state shame, reporting *p*-values and Cohen’s *d* effect sizes. We examined the correlation between nonverbal expressions of shame and self-reported state shame. We examined whether T2 nonresponders differed in terms of sociodemographic characteristics from responders.

To test our main moderation hypothesis, we used the bootstrap moderation method [54]. This method calculates the conditional effect of nonverbal expressions of shame on suicidal ideation for rural vs. urban participants, through bootstrapping, set at 5000 samples. All analyses were run using IBM SPSS 29.0 with PROCESS statistical program [54] We regressed suicidal ideation at T2 on rurality, nonverbal expressions of shame, the interaction term of nonverbal expressions of shame by rurality, controlling for suicidal ideation at T1 to investigate their individual contributions and potential combined effects on suicidal ideation at T2. Finally, we regressed suicidal ideation at T2 on rurality, self-reported shame, and the interaction term of self-reported shame by rurality, controlling for suicidal ideation at T1 to investigate their individual contributions and potential combined effects on suicidal ideation at T2. For all moderation analyses, we report the overall model significance and effect size (*f*^*2*^) as well as standardized coefficients and associated *p*-values for each predictor.

## Results

Participants from the rural sample were younger and more identified as non-Hispanic White compared to the urban sample (Table 1).

**Table 1:** Demographic information stratified by residence.

	Total Sample N (%)	Rural Maine Sample N (%)	Urban NYC Sample N (%)	Pearson Chi Square or t-test Comparing Rural vs. Urban Sample
Time 1 (T1)	133	68	65	
Time 2 (T2)	99	48	51	
Gender Identity				Chi Square = 0.06, *p* = 0.97
Man	34 (26)	18 (27)	16 (25)	
Woman	76 (57)	39 (57)	37 (57)	
Transgender Man	4 (3)	2 (3)	2 (3)	
Transgender Woman	3 (2)	1 (2)	2 (3)	
Nonbinary	19 (14)	8 (12)	11 (17)	
Additional gender category	11 (8)	6 (9)	5 (8)	
Sexual Orientation				Chi Square = 5.21, *p* = 0.39
Lesbian, gay, or homosexual	45 (34)	27 (40)	18 (28)	
Bisexual	58 (44)	29 (43)	29 (45)	
Queer	19 (14)	8 (12)	11 (17)	
Straight or heterosexual	1 (1)	1 (2)	0	
Something else or don’t know	11 (8)	4 (6)	7 (11)	
Race				Chi Square = 21.67***
White	105 (79)	66 (97)	39 (60)	
Black	11 (8)	4 (6)	7 (11)	
Asian	11 (8)	2 (3)	9 (14)	
Native Hawaiian or Pacific Islander	1 (1)	1 (2)	0	
American Indian or Alaska Native	2 (2)	0	2 (3)	
Something else	16 (12)	2 (3)	14 (22)	
Ethnicity				Chi Square = 10.27***
Hispanic or Latine	21 (16)	4 (6)	17 (26)	
Not Hispanic or Latine	112 (84)	64 (94)	48 (74)	
Age M (SD)	22.63 (7)	20.32 (4)	25.05 (8)	*t* = 4.36*** Cohen’s *d* = 0.76 (95% CI: 0.40, 1.11)
Range	18–53	18–40	18–53	

*Note*. Not all categories add to 100% as participants could select multiple identities per category. M is mean, SD is standard deviation. T1 is Time 1, T2 is Time 2. CI is Confidence Interval.

***p < .001

There were no significant differences in nonverbal expressions of shame by rurality [*t*(119) = 0.19, *p* = 0.85, *d* = 0.04] or self-reported state shame by rurality [*t*(129) = 1.24, *p* = 0.22, *d* = 0.22]. Intercorrelations between state shame, nonverbal shame, and suicidal ideation at T1 and T2 are reported in Table 2. Consistent with the literature, nonverbal expressions of shame were not associated with self-reported shame. Nonverbal shame and state shame (both at T1) were related to suicidal ideation at T2, while state shame at T1 was also related to suicidal ideation at T1 (Table 2).

**Table 2:** Correlations between state shame, nonverbal shame, and suicidal ideation at T1 and T2.

	1	2	3
1. State Shame T1			
2. Nonverbal Shame T1	0.00		
3. Suicidal Ideation T1	0.32***	0.03	
4. Suicidal Ideation T2	0.35***	0.20*	0.27***

Note.

* *p* < .05

***p < .001. T1 is Time 1 and T2 is Time 2 (3 months later). Numbers 1, 2, and 3 in top row denote variables listed in first column.

Additionally, nonresponding participants at T2 did not differ from responders at T2, thus the missing data does not appear to be confounded by location, shame, or suicidal ideation (Table 3).

**Table 3:** Means and standard deviations of state shame, nonverbal shame, suicidal ideation at T1 and T2 by region and differences between T2 responders vs. nonresponders within samples.

	Rural Maine Sample				Urban NYC Sample			
	Total	Response at T2	No Response at T2	*t*-test	Total	Response at T2	No Response at T2	*t*-test
	M (SD)	M (SD)	M (SD)	*p*-value	M (SD)	M (SD)	M (SD)	*p*-value
State Shame T1	1.80	1.67	1.53	-0.77	1.64	1.75	1.91	0.73
	(0.82)	(0.67)	(0.41)	*p* = 0.45	(0.63)	(0.80)	(.88)	*p* = 0.47
Nonverbal Shame T1	1.08	1.11	1.13	0.07	1.11	1.01	1.28	1.30
	(0.79)	(0.87)	(0.80)	*p* = 0.94	(0.85)	(0.73)	(0.92)	*p* = 0.20
Suicidal Ideation T1	2.43	4.65	4.29	-0.74	4.57	2.19	3.00	1.11
	(2.77)	(1.78)	(0.83)	*p* = 0.46	(1.62)	(2.65)	(3.03)	*p* = 0.27
Suicidal Ideation T2	1.33				0.65			
	(1.95)	---	---	---	(1.26)	---	---	---

*Note*. The columns under Rural vs. Urban Sample are specific to each respective sample. T1 is Time 1 and T2 is Time 2 (3 months later). M is mean, SD is standard deviation.

**Fig 1:**
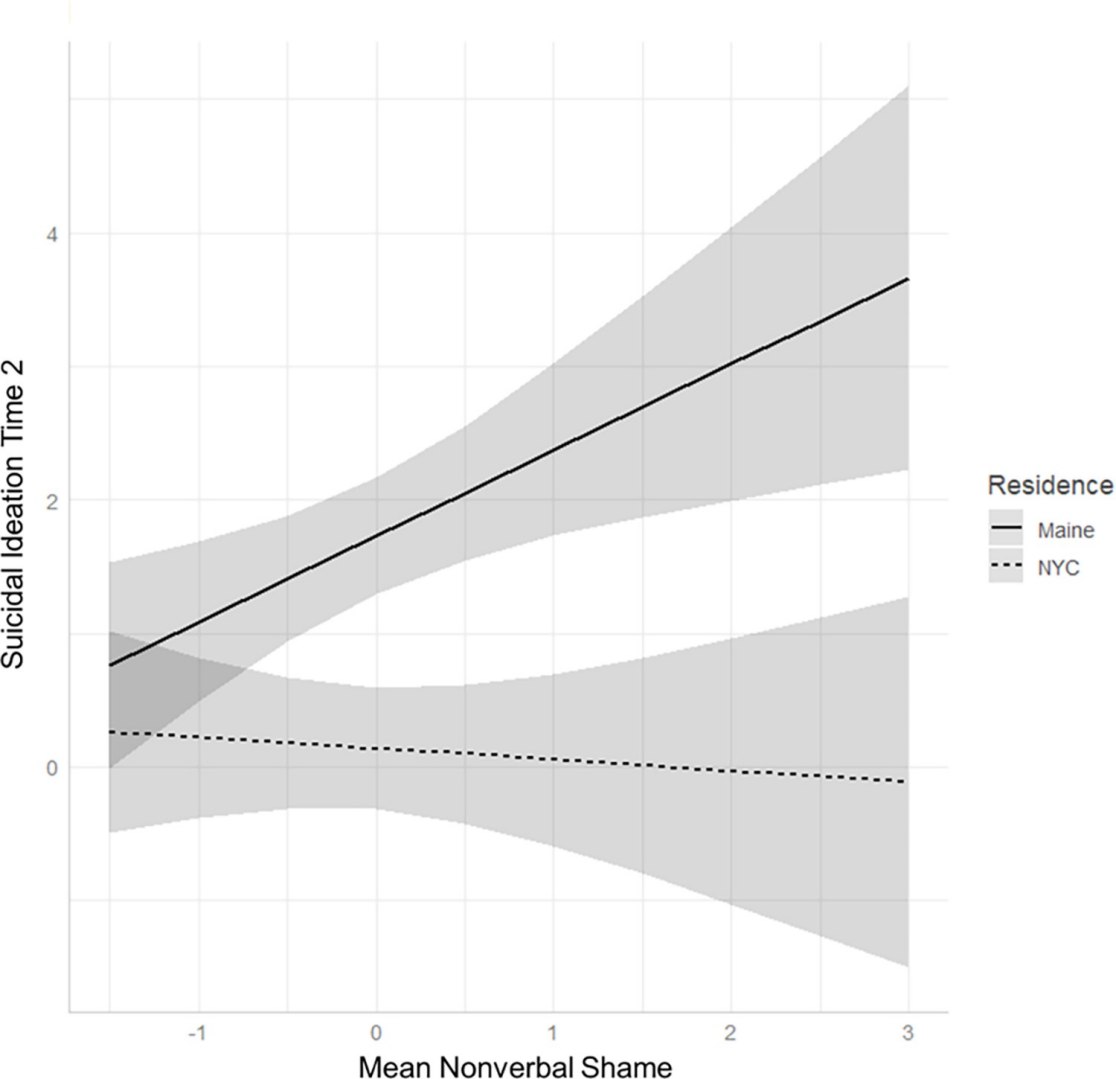
Regression plot showing relationship between nonverbal expressions of shame and suicidal ideation three months later at Time 2 (T2), controlling for Time 1 (T1) suicidal ideation, moderated by residence.

To test our main hypothesis, we regressed suicidal ideation at T2 on rurality, nonverbal expressions of shame, and the interaction term of nonverbal expressions of shame by rurality, controlling for suicidal ideation at T1. The overall model was significant, *F*(4, 87) = 9.35, *p* < .001, *f*^*2*^ = 0.43. Suicidal ideation at T1 (*B*_*std*_ = 0.45, *p* < .001) and rurality (*B*_*std*_ = 0.48, *p* < .001) were both significant predictors of suicidal ideation at T2, while nonverbal expressions of shame was not a significant predictor (*B*_*std*_ = -0.05, *p* = 0.70). These effects, however, were qualified by a significant nonverbal expression of shame by rurality interaction (*B*_*std*_ = 0.29, *p* = 0.02), such that higher intensities of nonverbal expressions of shame at T1 predicted increased suicidal ideation three months later (at T2) for rural (*B*_*std*_ = 0.64, *p* = .005) but not for urban LGBTQ individuals (*B*_*std*_ = -0.08, *p* = 0.70) (Fig 1). These same patterns remained when age and race/ethnicity were entered as covariates.

We also tested this model without controlling for suicidal ideation at T1. This model, *F*(3, 88) = 5.18, *p* = .002, *f*^*2*^ = 0.18, resulted in a similar marginally significant nonverbal expression of shame by rurality interaction effect (*B*_*std*_ = 0.64, *p* = .06) with higher intensities of nonverbal expressions of shame at T1 predicting higher T2 suicidal ideation for rural (*B*_*std*_ = 0.73, *p* = 0.004) but not for urban (*B*_*std*_ = 0.09, *p* = 0.71) LGBTQ participants.

These patterns were not replicated when *self-reported shame*, suicidal ideation at T1, and the interaction of *self-reported shame* and rurality were used to predict suicidal ideation at T2. While the overall model was statistically significant, *F*(4, 94) = 8.60, *p* < .001, *f*^*2*^ = 0.37, neither self-reported state shame (*B*_std_ = 0.29, *p* = 0.20) nor the interaction of self-reported state shame and rurality (*B*_*std*_ = 0.12, *p* = 0.69) significantly predicted suicidal ideation at T2. Rurality (*B*_std_ = 0.39, *p* < .001) and suicidal ideation at T1 (*B*_std_ = 0.40, *p* < .001) remained significant predictors of suicidal ideation at T2. These same patterns remained when age and race/ethnicity were entered as covariates.

## Discussion

Nonverbal expressions of shame appear to play a unique role in predicting suicidal ideation among rural LGBTQ individuals, but not among their urban counterparts. For rural LGBTQ individuals, more nonverbal expressions of shame predicted higher suicidal ideation three months later. Self-reported state shame did not uniquely predict suicidal ideation among rural or urban LGBTQ people. These findings are consistent with the minority stress model [6–8] and prior research that links shame to psychological distress [55] and suicide risk [56] and extends this literature by documenting the unique predictive power of nonverbal expressions of shame, especially among populations who experience structural level stigma.

The results of this study highlight the importance of considering cultural and social contexts when assessing the impact of shame on mental health outcomes within LGBTQ communities. Findings suggest that for those living in more rural areas where stigma may be more pervasive, embodied shame is closely tied to one’s mental health. In more urban settings, where stigma may be less pervasive and community support more available, the link between embodied shame and suicidal ideation is less apparent, highlighting the importance of social support, representation, and belonging [57].

Past work has similarly found a lack of relationship between self-reported shame and nonverbal expressions of shame [49]. We believe this might mean that self-reported shame is not always a valid indicator of state shame. As discussed in the introduction, for some, self-reported shame may be inaccessible and thus nonverbal expressions may be tapping into a subconscious experience of internalized stigma that self-reported shame cannot explain. This lack of a significant relationship between self-reported state shame and suicidal ideation further highlights the distinctive predictive power of nonverbal expressions of shame. We call for future researchers to continue understanding behavioral manifestations of stigma and health. Additionally, multi-method assessments of shame may allow for the most robust understanding of how stigma gets under the skin, embodied, understood, and impacts individuals’ cognitions, feelings, and behaviors.

In clinical settings, the novel association between nonverbal expressions of shame and increased suicidal ideation in rural settings underlines the need for clinicians to be attuned to subtle nonverbal cues and tailor interventions accordingly. Thus, identifying key behavioral manifestations of shame will allow for potentially earlier intervention, especially among populations who experience structural level stigma. Past work training clinicians to perceive their patient’s emotions more accurately has demonstrated promising results. For example, short discussion, practice, and feedback trainings have been shown to significantly increase clinician and laypeople’s accuracy in detecting other’s emotional states [58–60]. Additionally, artificial intelligence may be a promising assistive technology in recognizing shame expressions and prompting intervention by trained, affirming clinicians [61].

### Limitations

While the current study is the first to show the predictive power of nonverbal expressions of shame on suicidal ideation among rural and urban LGBTQ samples, this work is not without limitations. Future research with larger and more diverse populations are needed, including participants from other regions. The consideration of structural stigma as indicated by two states may not fully represent the experiences of rural and urban LGBTQ individuals nationwide. Future work should utilize more comprehensive measures of suicidal ideation. Future research should consider exploring shame within specific sexual and gender identities to gain a more nuanced understanding of its effects on suicidality and intersections with other marginalized identities.

### Implications

The study’s strengths lie in successfully measuring nonverbal shame both in person and through teleconferencing, revealing its association with changes in suicidal ideation among rural LGBTQ adults. Embodied shame is a modifiable risk factor for suicide prevention among LGBTQ people in more rural settings. These findings have significant implications for clinical practice, enabling mental health professionals to identify emotional distress more effectively and to modify interventions to support stigmatized populations. Study findings emphasize the urgency of understanding shame’s impact on LGBTQ peoples’ mental health, calling to action societal interventions that reduce stigma as well as interventions that promote regulatory strategies to foster acceptance and identity affirmation among LGBTQ individuals and specifically target shame such as ESTEEM therapy [62] or a self-compassion intervention [63].

## Conclusion

This research demonstrates the unique predictive power of nonverbal expressions of shame compared to self-reported measures, signifying the importance of recognizing and understanding nonverbal cues as potential indicators of mental health and wellbeing particularly in environments with higher levels of structural stigma. Given the rise in anti-LGBTQ policies and legislation in the US, especially in more rural locations [64], this work has implications for those who care for and support LGBTQ populations. By identifying suicide risk through behavioral manifestations of shame, close others and clinicians can affirm LGBTQ people’s identity, worth, and belonging, and potentially save lives.
